# Male competition reverses female preference for male chemical cues

**DOI:** 10.1002/ece3.7348

**Published:** 2021-03-09

**Authors:** Zorimar Vilella‐Pacheco, Lisa D. Mitchem, Vincent A. Formica, Edmund D. Brodie

**Affiliations:** ^1^ Department of Biology University of Puerto Rico—Arecibo Arecibo Puerto Rico; ^2^ Mountain Lake Biological Station and Department of Biology University of Virginia Charlottesville VA USA; ^3^ Department of Biology Swarthmore College Swarthmore PA USA

**Keywords:** *Bolitotheruscornutus*, chemical cues, female choice, male, male competition

## Abstract

Females must choose among potential mates with different phenotypes in a variety of social contexts. Many male traits are inherent and unchanging, but others are labile to social context. Competition, for example, can cause physiological changes that reflect recent wins and losses that fluctuate throughout time. We may expect females to respond differently to males depending on the outcome of their most recent fight. In *Bolitotherus cornutus* (forked fungus beetles), males compete for access to females, but copulation requires female cooperation. In this study, we use behavioral trials to determine whether females use chemical cues to differentiate between males and whether the outcome of recent male competition alters female preference. We measured female association time with chemical cues of two size‐matched males both before and after male–male competition. Females in our study preferred to associate with future losers before males interacted, but changed their preference for realized winners following male competitive interactions. Our study provides the first evidence of change in female preference based solely on the outcome of male–male competition.

## Graphical abstract

In forked fungus beetles, the act of competing changes male odors in a way that alters how females perceive them. Before males compete, females prefer the scent of males who are eventual losers, but after competition, females switch to prefer winners. In the wild, all males may have access to females at different times depending on the recency of competition.

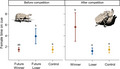

## 1 INTRODUCTION

Sexual selection operates through a dynamic interplay between intra‐ and intersexual selection (Hunt et al., 2009). In systems where males gain access to potential mates via intrasexual competition, females tend to be the choosier sex and often assess mate quality by assessing specific male characteristics associated with success in competition (Candolin, 1999; Filice & Dukas, 2019; Hunt et al., 2009; Wong & Candolin, 2005). Competitive interactions can increase a male's opportunity to mate with females by excluding or outcompeting other, less dominant, males (Andersson et al., 2002). Although traits associated with competition success are usually a good indication of male quality (Ditchkoff et al., 2001; Rantala & Kortet, 2004; Zahavi, 1975), dominance may be accompanied by aggressive behaviors or deceptive signals that can negatively affect female fitness (Kiyose et al., 2015; Moore et al., 2003; Sih et al., 2014). These fitness effects associated with male competitive traits may consequently alter female preference (Moore et al., 2001; Sih et al., 2014). The outcomes of female choice, however, can be difficult to determine in systems where males are highly aggressive and exclude other males (Wong & Candolin, 2005); nevertheless, the role of female preference in sexual selection can be determined in systems where females—either through pre or postcopulatory mechanisms—control male mating success (Firman et al., 2017; Wong & Candolin, 2005).

One way females can detect and assess quality of potential mates is through pheromones (Johansson & Jones, 2007), and cuticular hydrocarbons in insects (Ivy et al., 2005; Roux et al., 2002; Thomas & Simmons, 2009). Pheromones and cuticular hydrocarbons can be used as indicators of mate quality and social status, and aid in the assessment of direct or indirect benefits related to the mating process (Baruffaldi & Andrade, 2015; Harari et al., 2011; Johansson & Jones, 2007; Steiger & Stökl, 2014). For example, male *Nauphoeta cinerea* competition status is associated differences in pheromone and cuticular hydrocarbon profile (Moore et al., 2001; Roux et al., 2002), and females use these olfactory signals to identify subordinate males, which they prefer to mate with over violent, dominant males (Moore et al., 2001, 2003). Chemical cues sometimes provide information independently of direct interaction when individuals deposit cues on substrates or release into air (Larsson, 2003).

Signal traits assessed during mate choice are often condition‐dependent. Some male traits, such as body size, remain static throughout the breeding season whereas social status and other sexual signals can fluctuate through time and depend on the outcome of recent interactions. Specifically, frequent winners are more likely to engage in and win aggressive encounters, whereas losers tend to be more submissive and avoid aggressive interactions (Hsu et al., 2006; Hsu & Wolf, 1999). Experimental studies show that changes in the internal state of an organism—including changes in nutritional state and hormone levels—lead to changes in the expression of sexual signals (Reviewed in: Vitousek et al., 2014). Furthermore, both the internal state and the behavior of individuals can be affected by social context. Aggressive encounters can alter the endocrine state of individuals (Earley & Hsu, 2008); bursts of stress‐induced hormones, for example, can intensify aggression, while prolonged high levels of these hormones have the opposite effect (Mikics et al., 2004; Øverli et al., 2002). Male competition thereby has the potential to alter chemical signals (Candolin, 1999; Rhodes & Schlupp, 2012; Setchell & Dixson, 2001), which are often dually used for competition and female choice (Johnstone, 1995; Martín & López, 2009; Tarof et al., 2005). The question remains, are males chosen because of their inherent and unchanging phenotype, or can female choice be influenced by temporally fluctuating social status?

In this study, we examined the behavior of *Bolitotherus cornutus* (forked fungus beetles) to ask: (a) Do females perceive males through substrate born chemical cues?, (b) Do females choose to associate with the chemical cues of winning males?, and (c) Do female choose to associate with male chemical cues based on their interactivity? *Bolitotherus cornutus* are a tenebrionid beetle in which males are distinguished by the presence of elaborate horns, which they use in competition for access to females (Conner, 1988, 1989). Usually the larger, more aggressive males with long horns win opportunities to mate and typically choose to court bigger females (Conner, 1988, 1989; Formica et al., 2016; Mitchem et al., 2019). Males are observed in male–male combat both in the presence and absence of females (Conner, 1988). Females appear to have little control over which males court her, yet they do control the ultimate decision of who to copulate with (Brown et al., 1985). Females have an anal sternite (a hard covering on the ventral surface of the terminal abdominal segment) that acts as a lock system giving the female control over copulation (Conner, 1988), and may use chemical cues during courtship to determine whether copulation should proceed. In this system, larger males have been considered to be of higher quality with more access to females (Conner, 1988; Formica et al., 2016; Mitchem et al., 2019), but previous work done in controlled experiments suggests that females do not necessarily prefer larger males (Brown & Bartalon, 1986; Brown et al., 1985). These studies, however, took place in the absence of male competition. The outcome of competition may affect who females prefer to associate with.

Despite rising interest in the mechanisms underlying chemical signaling, surprisingly little is known about chemical communication in *B. cornutus*. *Bolitotherus cornutus* produce a defensive chemical secretion when disturbed and that these chemical secretions are diet‐based (Conner et al., 1985; Holliday et al., 2009; Tschinkel, 1975), but no previous studies determine how pheromones or other chemical signals, if any, aid in communication among *B. cornutus* in competition and mating interactions. We conducted a sequence of two female choice trials to determine whether *B. cornutus* females prefer to associate with the chemical signals of winning males. First, we performed female choice trials to test whether female beetles use substrate born chemical cues to detect and distinguish between males. Following the first female choice assays, we used the same male beetles from which we collected scent to conduct male interaction trials and determine which males were winners in male competition. Lastly, we performed a second set of female choice trials using the same male pairs to test whether females altered their preference (i.e., female choice, given that female preference is restricted to our experimental conditions) for winning or losing males.

## 2 METHODS

### 2.1 Study species

We collected a total of 75 (50 male and 25 female) beetles from a large metapopulation near Butt Mountain, Virginia, in June 2019. We housed subjects individually in small, plastic containers (5 cm × 2.5 cm × 5 cm) under natural light conditions (14.5:9.5 hr light:dark cycle ± 17 min) and room temperature (22 ± 3°C) at the Mountain Lake Biological Station (Salt Pond Mountain). Containers included a small amount of mulch over a layer of plaster of Paris to help maintain a humid environment and mimic natural substrate. We added a small piece of *Ganoderma tsugae* (a polypore fungus) fruiting body as a food source and provisioned water ad libitum. Beetles remained in isolation in their containers for 12 days before trials began. All individuals were assigned a unique ID by adding colored dots to their elytra using nontoxic Testors^®^ Enamel paint. We recorded elytra length to the nearest 0.01 mm from images taken with a flatbed scanner (Epson Perfection V600 Photo) in ImageJ (Abramoff et al., 2004; Formica et al., 2012). Age and reproductive status are impossible to determine from wild‐caught *B. cornutus*, so we did not control for the age or experience of our individuals.

### 2.2 Video recording

We assayed behaviors in a dark, temperature‐controlled room at 19 ± 2°C using infrared lights to enhance visibility in video recordings. Female choice trials were video recorded for 2 hr, and male competition trials were recorded for 4 hr. We placed a Canon PowerShot G1 X digital camera approximately 1m above the arena to record behaviors by taking a photograph every five seconds for the duration of the trial using a Neewer© LCD digital shutter‐release remote control. We processed these images into a 5‐min time‐lapse video using FFpeg software (Version beld1d234). We then scored the initiation and duration of behaviors using Inqscribe^®^ transcription software (version 2.2.4; Mitchem et al., 2019).

### 2.3 Overview of experimental design

**FIGURE 1:**
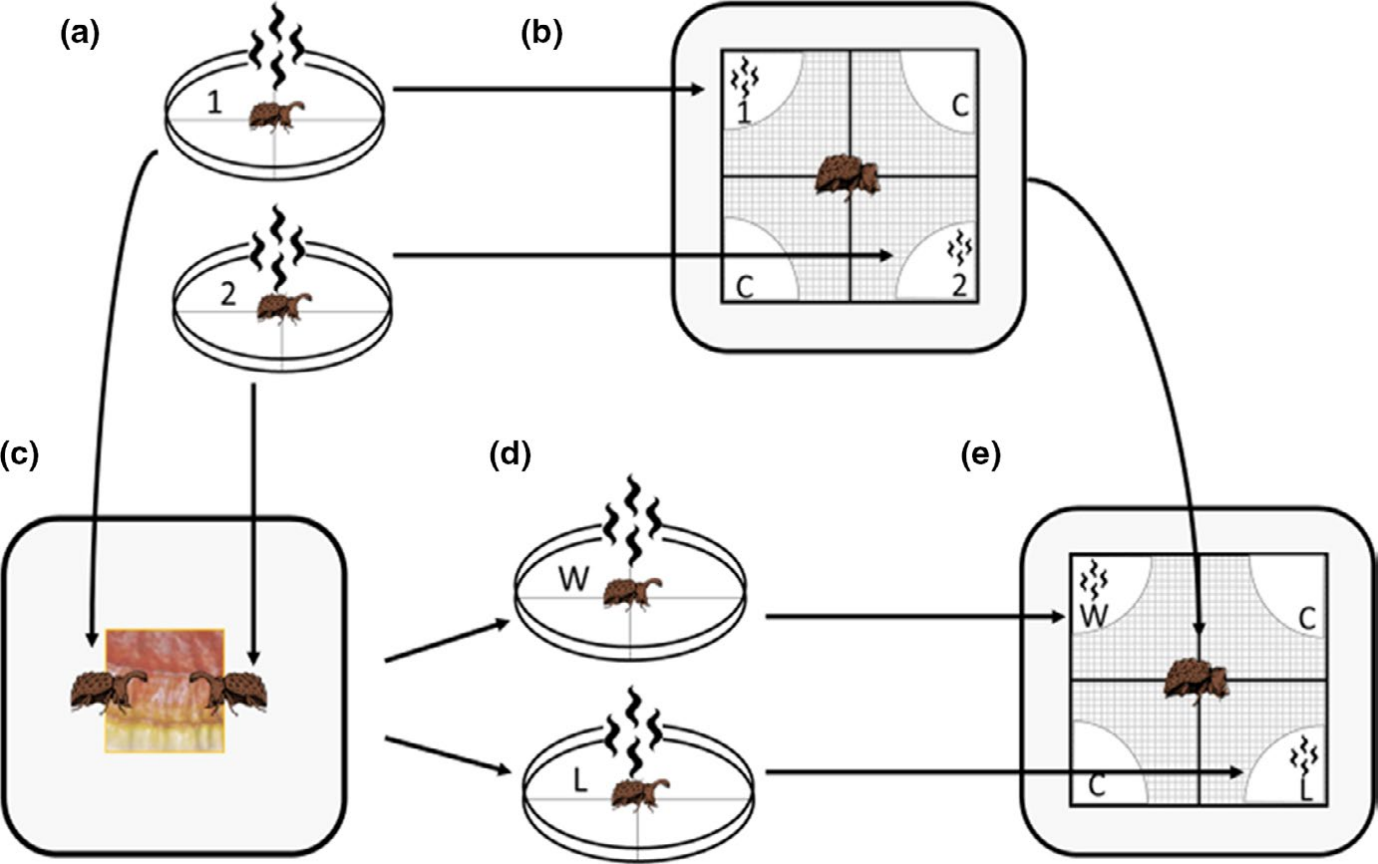
Scheme of experimental design. (a) First, we collected the chemical cues of two size‐matched males in Petri dishes lined with filter paper. (b) Second, we exposed females to the chemical cues of both males and two control filter papers simultaneously and recorded her movements and exploratory behaviors. (c) Third, we placed the same male pairs in male–male competition trials to determine winners (W) and losers (L). (d) Fourth, we immediately collected chemical cues from the same males following competition trials. (e) Fifth, we performed a second set of female choice trials with the new posttrial male cues

We conducted two female choice trials to assess if preference changes following male competition. We performed female choice and male competition assays in the following order (Figure 1): First, we provided females with the chemical cues of two size‐matched males prior to competition. Then, we placed those same males in a competition trial to determine the winner and loser of the pair and collected male chemical cues immediately posttrial. Finally, we performed a second set of female choice trials where females were exposed to the posttrial cues of the same male pair. This testing sequence was dictated by the goal of detecting whether females perceive differences in chemical cues resulting from male contests. Males could not be allowed to interact first and subsequently be treated as presenting an interaction‐free cue. Unfortunately, this constraint of the treatment did not allow us to control for potential order effects. The details for each set of behavioral trials are specified further in the corresponding sections.

### 2.4 Chemical cue collection

We collected chemical signals from males by placing individuals in small Petri dishes containing four equal, triangular pieces of a clean filter paper disk (50 mm diameter) for a period of 24 hr (Figure 1a). To prevent signal deterioration, pieces of filter paper remained inside Petri dishes and were used within 2 hr after removing males. We modified this method from Kortet and Hedrick (2005). The colored ID was not visible in infrared video recordings; therefore, we added an additional white dot to the elytra of a randomly chosen male 24 hr prior to male scent collection.

### 2.5 Precompetition female choice assays

Female choice trials were recorded for a period of 2 hr. We collected fresh male chemical cues before each female choice trial. We then presented females with the chemical cues of two size‐matched males and two control (i.e., clean filter paper) cues and allowed her to associate freely with the cues of either male or unexposed controls. Female choice arenas consisted of a plastic box (17 cm L × 15 cm W, 11.14 cm H) with a layer of white gridded paper. We taped filter paper cues to the grid paper equidistantly around the center of the arena using clear tape. We placed paper containing different male cues diagonally across from each other in every trial. Control cues were placed in between male cues and diagonally across from each other. Treatments were placed 4.5 cm apart and at least 2 cm away from the edges of the arena (Figure 1b). Female beetles were released in the center of the arena after an acclimation period of at least 4 min under a plastic vial. We recorded the number of times females initiated contact with each filter paper and the duration of time females were in physical contact with each filter paper. We used total time a female spent physically touching the different pieces of filter paper relative to her total time spent active as a proxy for female choice (Brown et al., 1985; Kortet & Hedrick, 2005). Further, filter paper covered only approximately 8% of the area in the arena; thus, the total time spent on chemical signals did not necessarily equal the total time of the trial. We modified this method from (Kortet & Hedrick, 2005).

### 2.6 Male interaction trials

The purpose of our male competition trials was to induce winning and losing status in size‐matched male pairs. We paired size‐matched males in dyadic trials to determine winners and losers of intrasexual competition. Size‐matching males within 0.05 mm allowed us to assess the effects of winning status independent of differences in male size, which is known to correlate with competition status (Mitchem et al., 2019). We embedded a 5 cm × 5 cm square piece of *Ganoderma tsugae* in the center of the arena (described above) as a resource for the beetles to compete over. Trials began with each male placed on an opposite side of the *G. tsugae* square and ended after 4 hr of recording. Competition duration in *B. cornutus* has been previously documented as lasting from a few seconds to about half an hour (Brown, 1980; Mitchem et al., 2019), so our 4‐hr trial period allowed us to observe the totality of competition from onset to completion. The 4‐hr period also allowed for reinforcement of winning and losing statuses due to repeated postcompetition encounters during the trial time. Male competition did not isolate winning status as the only differences between males. Males may also differ in expression of behaviors. We recorded the initiation and duration of the aggressive and nonaggressive behaviors using procedures described in Mitchem et al. (2019). We also recorded the number of times each male ended an interaction, defined as leaving the area before its competitor. The beetle who ended more interactions was assigned “loser” status. No beetles died during experimental trials.

### 2.7 Postcompetition female choice trials

Following male competition trials, we conducted a second female choice assay where we presented females with the new chemical cues of the same two males to determine whether her preference changed postcompetition. The second female choice trials began 26 hr after completion of the male competition trials. We immediately placed males on new filter paper to collect postcompetition chemical cues for 24 hr. After 24 hr of cue collection, we constructed new female choice arenas, a process that took 2 hr. Female choice trials then proceeded the same as the first round.

### 2.8 Statistical analyses

We first asked if females spent more time on filter paper than expected by chance. Filter papers occupied 8% of our total arena spaces, so our null expectation was females spend 8% of their 2‐hr trial time (9.8 min). We tested this assumption using a one‐sample *t* test comparing the duration of time females spent on any filter paper (male cue and control) to the null expectation of 9.8 min. Next, to test whether females differentiated between male cues and control filter paper, we assessed the effects of male cue versus control cue on female choice using a generalized linear mixed model with a Poisson distribution where duration of time and counts of contact with cues were our controls in two separate models. We included the counts and durations for females in both before and after male competition trials for this model. We included filter paper treatment (male cue vs. control), trial date (before or after male–male competition trials), and their interaction as fixed effects. We included female ID as a random effect.

To determine whether females distinguished between males and demonstrated a preference for winning or losing males, we grouped competitors into “future winners” and “future losers” before interaction and winners and losers after interaction. We assessed the effect of male competition status (both pre‐ and postcompetition) on the duration of time spent on each filter paper using a zero‐inflated generalized linear mixed model with a Poisson distribution. We included cue type (winner, loser, or control), trial experience (precompetition and postcompetition trials), and their interaction as fixed effects. We needed to standardize the proportion of time spent on each cue type because we presented females with one winner, one loser, and two control cues. To account for this difference in numbers of each type of cue, we pooled time on both control papers and divided that total by two. We included female ID as a random effect. We used pairwise comparisons of estimated marginal means to test for differences among winner, loser, and control filter papers.

Finally, we asked if females preferred cues of males based on their overall interactivity levels, specifically asking if females preferred more interactive males. We used counts of initiated behaviors with male competitors as our behavioral phenotype for each male. The number of initiated interactions is a repeatable trait (Mitchem et al., 2019), and likely indicative of true differences in interactive behaviors among males. We used a zero‐inflated generalized linear mixed model with identical fixed and random effects from the winner versus loser model. Here, we used the number of initiated behaviors in male competition trials, trial experience, and their interactions as fixed effects. Two females were excluded from all data analyses because they crawled under the experimental arena substrate during their trial, and one additional female was excluded because she remained inactive during the entire 2‐hr trial.

All statistical analyses were carried out using R v.3.6.0. We used the “lmer” package in R for our generalized linear mixed models (Bates et al., 2015), and the “glmmTMB” package for our zero‐inflated generalized linear mixed models (Brooks et al., 2017). We assessed the significance of our models with type 3 Walds *χ* ^2^ test using the “car” package in R (Weisberg, 2019). We compared estimated marginal means using the “emmeans” package (Lenth et al., 2020). We tested out model uniformity, zero inflation, and dispersion using the DHARMa package in R (Hartig, 2016).

## 3 RESULTS

**FIGURE 2:**
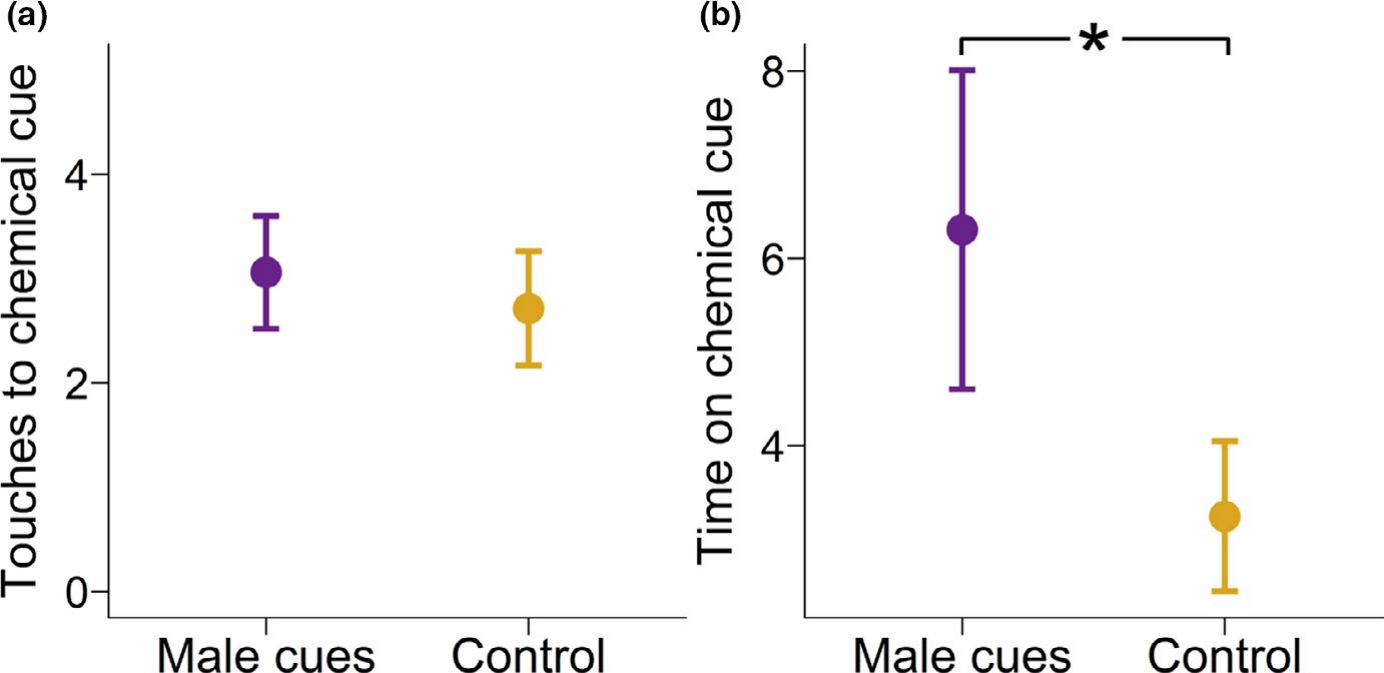
(a) Mean (±*SE*) count of initiated touches to control filter paper and male chemical cue filter paper combined from both before and after male competition trials. (b) Mean (±*SE*) duration in minutes females spent on control filter paper vs. male chemical cue filter paper combined from both before and after male competition trials

Females spent an average of 10.35 min of the 2‐hr trial physically touching filter papers in both pre‐ and postcompetition trials. Compared to our null assumption, females interacted with any filter paper in a proportion that was expected by chance (*t* = 0.29, *df* = 48, *p* =.77). Females did not differ in the number of touches they initiated for either control or male filter paper (*χ* ^2^ = 0.09, *df* = 1, *p* = .77; Figure 2a). However, females, did spend significantly more time on the filter paper of male cues compared to control cues (*χ* ^2^ = 14.17, *df* = 1, *p* < .001; Figure 2b). The average number of touches to any filter paper (male cue or control) was slightly greater in the postcompetition trials (*χ* ^2^ = 3.84, *df* = 1, *p* = .05) in which females performed an average of 3.92 touches in the postcompetition female choice trials compared to 1.92 touches in the precompetition trials. Females did not differ in their duration of association time with any filter paper before (Figure 1b) or after (Figure 1e) male competition trials (*χ* ^2^ = 0.02, *df* = 1, *p* = .88).

**FIGURE 3:**
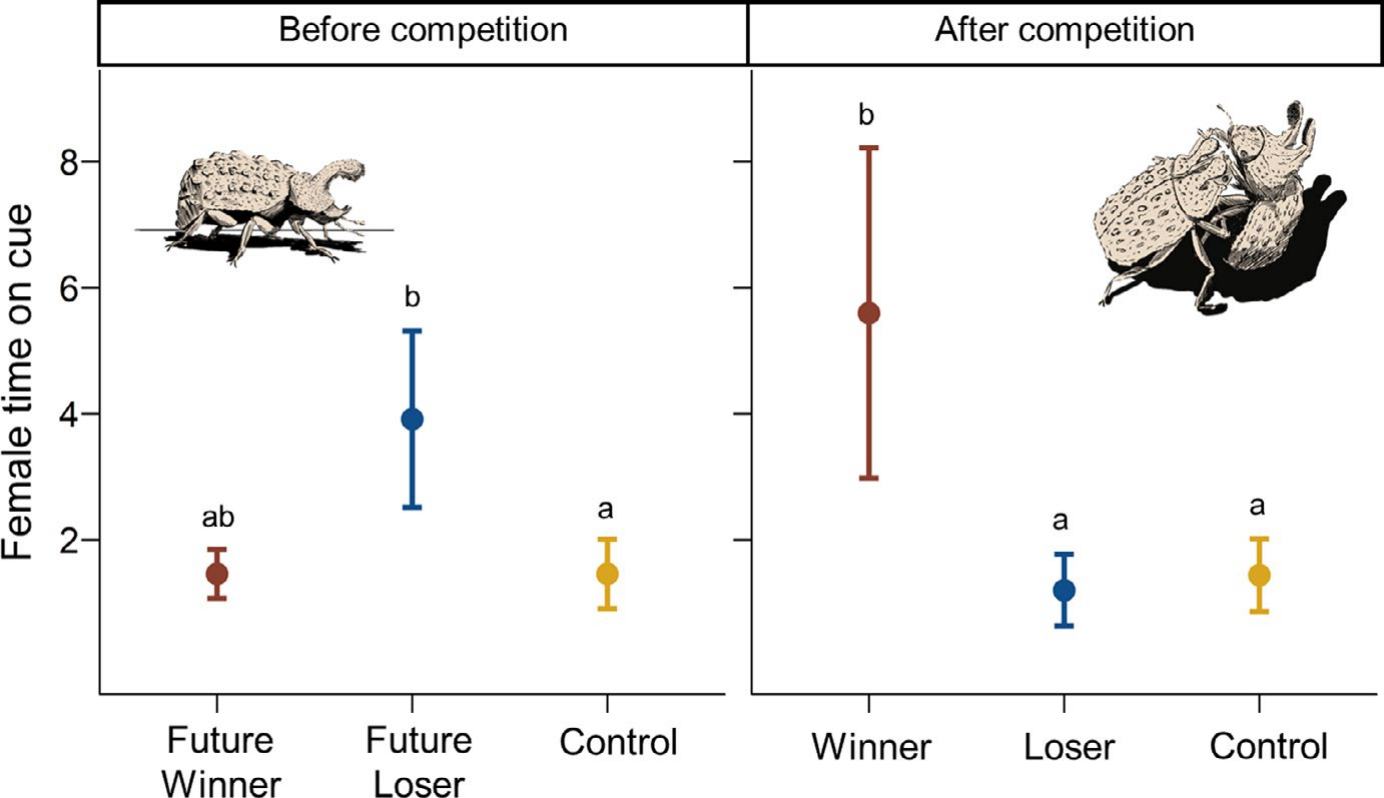
Mean (±*SE*) minutes females spent on filter paper of future winning vs. future losing male chemical cues before male–male competition trials (left panel) and realized winning and losing males after male–male competition trials (right panel). Future winner/loser and behavior status was retroactively assigned after analysis of male–male competition videos. Drawings by Miles Bensky

**FIGURE 4:**
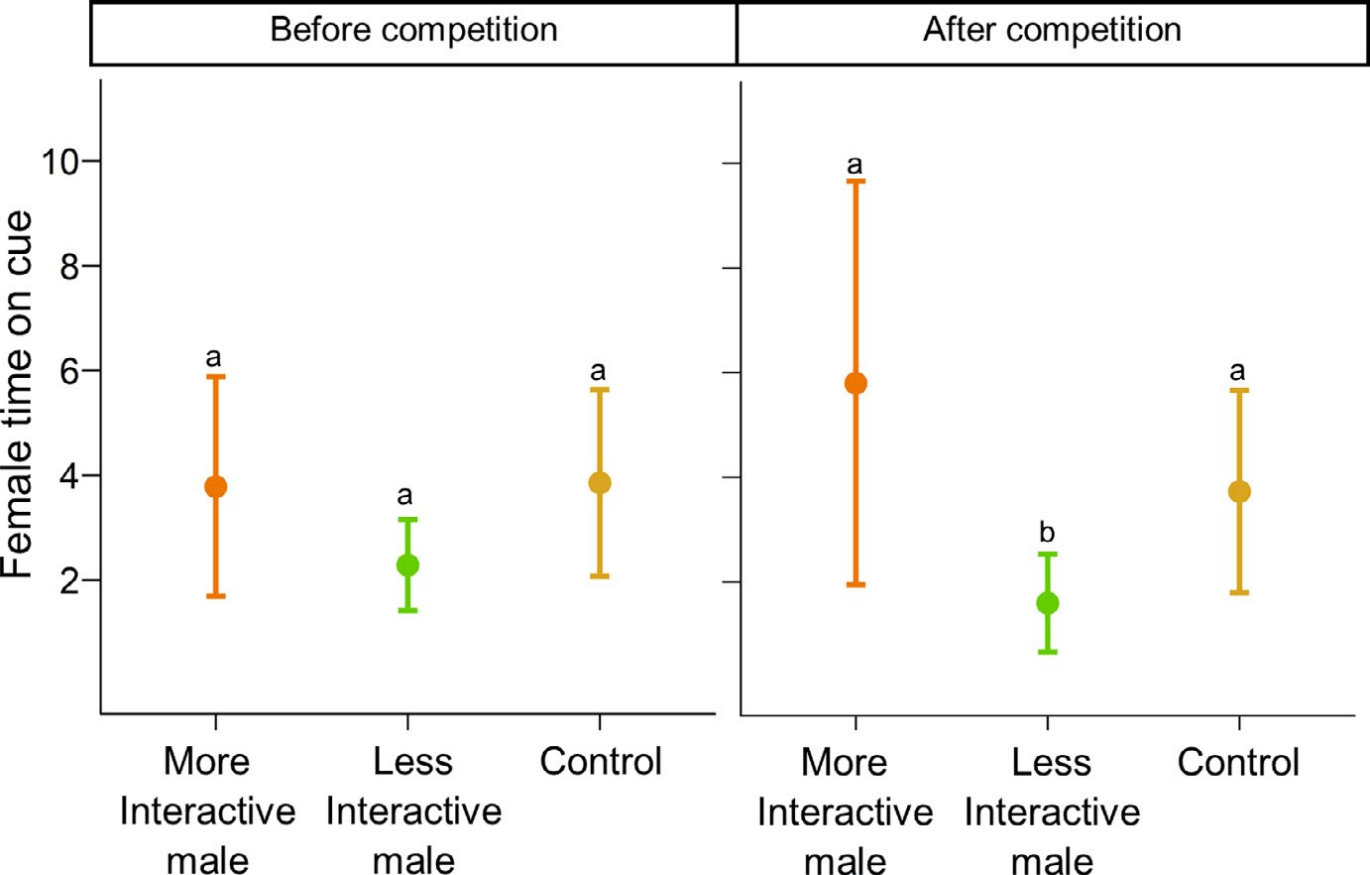
Mean (±*SE*) minutes females spent on filter paper of males who initiated more interactions in male–male competition trials vs. males who initiated less interactions for before male–male competition trials (left panel) and after male–male competition trials (right panel). The number of initiated behaviors for both males within each trial was retroactively assigned after analysis of male–male competition videos

Females changed how much time they associated with the cues of winning and losing males after male–male competition trials (male status by trial experience interaction: *χ* ^2^_(male status × pre‐ vs. post‐competition)_ = 31.0513, *df* = 2, *p* < .001; Figure 3). Females did not spend more time interacting with future losers compared to future winners (estimate = −0.40, *SE* = 0.247, *p* = .59) but did spend more time interacting with future losers compared to control papers (estimate = −0.74, *SE* = 0.226, *p* = .02) before male–male competition. Females did not differ in their association time with future winner cues compared to control papers (estimate = −0.37, *SE* = 0.267, *p* = .79). When exposed to chemical cues collected after male–male competition trials, however, females spent significantly more time on the filter paper of winning males compared to loser males (estimate = 1.51, *SE* = 0.243, *p* = <.001). Females did not differ in their association time between loser males and control papers after male competition trials (estimate = 0.04, *SE* = 0.283, *p* = 1.00). Females did not prefer to associate with males based on how much they initiated interactions before male competition but changed their preference for more initiative males, who are more likely to win, after competition (*χ* ^2^_(male behaviour × pre‐ vs. post‐competition)_ = 8.06, *df* = 2, *p* = .02; Figure 4).

## 4 DISCUSSION

The outcome of intrasexual competition can alter the way males are perceived by their potential mates. Females in our study preferred chemical cues of males based on their status following competition. Females spent more time interacting with future losing chemical cues compared to control, but interacted equally with future winners and loser before competition. Females then switched their preference to cues of realized winners after competition. The change in preference following male–male competition indicates that competitive outcomes themselves may be an important driver for female choice.

Certain male features can directly influence both the outcomes of male competition and female choice including body size, armament size, and level of interactivity. We size matched males in our study to remove the opportunity for size differences to drive female choice, but male pairs may have differed in other morphology and behaviors. First, males differed in horn length, which is strongly, but not perfectly, correlated with body size (Conner, 1988). Our findings, however, suggest that females do not choose male chemical cues associated with horn length. Females chose between the same pair of males before and after competition; horn size did not change from trial to trial, but female preference did. Males also differ in their intrinsic behaviors. Winning males were frequently the more interactive males, or individuals who initiated more behaviors with their competitors. Females in our study preferred to associate with overall interactive males only following male competition, meaning females chose based on competition outcomes and not the inherent interactive phenotypes of males.

Females in our study may recognize the chemical cues of aggressive and nonaggressive behavioral types and show a slight, nonsignificant preference to associate with the nonaggressive, subordinate male cues before male competition occurs. Chemical cues associated with male quality may be difficult to assess before competition, leading to a lack of observed female preference between future winner and loser chemical cues before competition. Females, however, preferred future losers over control cues, which we interpret as a signal for potential preference in future losing males. Then, competition may induce changes in chemical cue composition that alter the way winners and losers are perceived by females. Females may benefit from selecting males that maximizing fecundity and offspring viability (Byers & Waits, 2006; Kiyose et al., 2015; Milinski & Bakker, 1990; Moore et al., 2003). Behavioral dominance is often associated with high immunocompetence and production of offspring with “good genes” (Hill, 1991; Hill & Montgomerie, 1994; López et al., 2002; Penn & Potts, 1998).

Three possible scenarios could alter male chemical signals following competition. First, either winning or losing males may produce different chemical cues following competition. Under this scenario, females are selecting for males based on new chemical phenotypes after competition compared to those before. Competition may stimulate the production of new or enhanced chemical components in winning males that are attractive to females or less attractive, possible stress‐induced, chemical components in losing males (Salvador & Costa, 2009). While no previous work documents change in chemical cues following competition, a similar phenomenon in display coloration has been noted in sticklebacks (Candolin, 1999). Brighter coloration of mating displays following competition is associated with winning male competitions, and winning males are preferred by females (Candolin, 1999). Competition may induce a stress response in losing males that makes them less attractive to females (Leary & Baugh, 2020). Chronic and acute stress responses reduce fitness and male attractiveness (Breuner et al., 2008; Creel, 2001). For example, increased level of the stress hormone corticosterone in male green treefrogs during male–male competition compromises male attractiveness by relocating energy from courtship behavior to survival (Leary & Crocker‐Buta, 2018). To test whether chemical cues are changed or enhanced following competition, we suggest analyzing individual male chemical cues before and after male competition to determine the specific pheromonal changes occurring during competition.

Alternatively, chemical cues may be transferred between males during male competition. Losing males may transfer a cue to winning males during combat, therefore explaining why winning males are preferred in postcompetition trials. However, given this scenario, we would expect losing and winning males to be equally preferred by females in postcompetition trials. Winning males may also apply an unattractive cue to losing males that masks their previously attractive cues. Given this explanation, we would predict females to avoid interactions with losing males. However, if winning males would apply an unattractive cue it is likely that remnants of the cue would still be perceived on themselves making them unattractive as well. Instead, our females interacted at a random frequency with losing males and preferred association with winning males after the competition (Figure 3). Countermarking occurs when individuals compete via chemical signals to ensure that their own scent masks any previous males’ scent marks and may influence female preference in favor of the male who conceals their competitor's cue (Fisher et al., 2003; Rich & Hurst, 1998, 1999). However, previous studies on countermarking examine urine‐based chemical cues for territory determination in mammals (Fisher et al., 2003; Rich & Hurst, 1998, 1999). There are no previously documented instances of countermarking or the application of unattractive chemical cues directly to opponents via bodily contact. To test whether countermarking explains changes in female preference, we suggest a combination of visual and chemical choice trials. First, allow females to interact freely with two males, then pair males in competition trials and cross‐transplant their chemical cues. Finally, allow females to reassess male visual and chemical cues.

Lastly, the switched preference for realized winners may be because females recognize odors of fungal resources collected by winning males. Winning males may gain more access to resources and therefore collect more fungus scent. While this scenario is possible, territoriality is not documented in *B. cornutus* and unlikely to affect female choice in wild populations (Brown, 1980; Conner, 1989). *Bolitotherus cornutus* live, feed, and reproduce on the fruiting bodies of wood‐decaying shelf fungi (Lile, 1956). Multiple males are often observed in close proximity while not engaging in aggressive behaviors the same fruiting body (Brown, 1980; Formica et al., 2012). All males to some degree have access to the same fungal resources on a given log making it unlikely that female choice is based on fungal odors (Conner, 1988). Nevertheless, the possibility of female preference based on intensity of fungal odor should be explored in the future and can be tested by placing males in competition trials that lack fungus resource.

The necessity of a fixed order in our experimental design means that we cannot completely rule out the possibility of order effects. Females could have haphazardly chosen to associate with one male in their first trials, then reversed their choice and associated with the opposite male in the postcompetition trials. A switch in preference might be expected if females respond to a lack of positive feedback (i.e., courtship) from their initial choice and so direct their attention to alternative male. The potential for such order effects to alter our main interpretations should be further explored. One way the potential order effects can be explored is to conduct a parallel experiment where females choose between the chemical cues of two size‐matched males at two different time points with no male competition trials. Results of female preference can then be compared between females given males with and without a competition context.

Social context extends beyond the immediate surrounding behavioral phenotypes to include the past experiences of social partners (Filice & Dukas, 2019; Hsu & Wolf, 1999; Oliveira et al., 2009; Vedenina & Shestakov, 2018). Previous interactions can perpetuate dominant/subordinate relationships, and ultimately affect who has access to mates (Hsu & Wolf, 1999; Oliveira et al., 2009, 2011). Our results indicate that chemical cues play a role in determining which males are preferred by females and suggest that preference for certain males may change based on past male experience. A male who has recently lost a competitive interaction will be assessed differently than that same male before competition, possibly resulting in opposite outcomes depending on the context. Winner–loser effects, where male competition outcome is dependent on previous competition experience, have been documented in a variety of species (Hsu & Wolf, 1999; Mesterton‐Gibbons et al., 2016), and these effects have been associated with hormonal changes in males (Oliveira et al., 2009). We might expect female choice to follow the direction of male chemical composition changes following competition to also be widespread across taxa.

Experienced‐based chemical communication may have major implications for the use of space, information transfer, and social organization. Our experiment shows that physical substrate can carry chemical cues with important social information in the absence of the individuals producing those cues. Male chemical marks may provide a record, and reliable indicator, of competitive ability (Rich & Hurst, 1999). Such chemical displays can then serve as advertisement for the attraction of potential mates as well as a challenge for sexual competitors (Johansson & Jones, 2007). We show that females can differentiate between winning and losing males. If females can also differentiate between males in nature, then we might expect females to associate with preferred winning males (Kodric‐brown & Nicoletto, 2001). Male sexual or competitive displays may also attract or repel competing males, resulting in agonistic encounters that can affect female choice and selection for male traits (Gosling & Roberts, 2001). The resulting space use and social organization within a population is then, in part, determined by the chemical phenotypes of the surrounding individuals.

Our results offer a potential explanation for why traits associated with losing males are maintained in wild populations. Traits associated with winning males should persist and increase within a population due to directional selection but directional selection on male competitive traits is not always observed in the wild (Hunt et al., 2009; Moore & Moore, 1999). Differences in female preferences across social contexts provide one explanation for why selection does not always favor these winning males. Males with traits that beget a tendency to lose battles may receive access to mates via female preference when competition is absent, whereas males with the tendency to win are preferred by mates immediately following competition. Frequency of competition is rarely constant across populations and can be spatially variable (Hunt et al., 2009). This fact means losing males may have the opportunity to attract mates in contexts where competition is low or absent. The resulting balancing selection could then lead to maintenance of traits associated with losing males.

The timing and outcome of competition are an important determinant of female choice. Male–male interactions change chemical composition in a way that reverses female preference, and the timing could impact social organization and maintenance of multiple male phenotypes within a population. Future work is needed to determine whether preference for chemical cues aligns with actual female mate choice in *B. cornutus*. Though controlling for order effects was not possible with this experimental design, measuring female preference both before and after male competitive interactions allowed us to demonstrate that competition can alter chemical cues in a way that changes female preference for one male over another. While our study did not determine the specific chemical changes that alter female preference, identifying the source of this information is an important next step. We also recommend additional studies that examine the potential for effects of testing order and chemical transfer between males during combat on female choice.

To our knowledge, we are the first to document change in female chemical preference for chemical cues following male competition. Previous studies assess female preference only following male competition (Darragh et al., 2017; Moore et al., 2001; Rich & Hurst, 1999), or in the absence of competition (Kortet & Hedrick, 2005; Moore & Moore, 1999). We show that it is not only the inherent phenotypes of individuals that determine female preference, but the process of competition itself that influences the outcome of potential mating decisions. Context and past experiences will influence an individual's potential for reproductive success. Overall, we would expect the interplay between experience of male competition and female choice to allow for the persistence of multiple male phenotypes within a population.

## 5 ETHICS STATEMENT

No licenses were required for the collection or use of forked fungus beetles in Pembroke, Virginia.

## CONFLICT OF INTEREST

The authors declare no competing interests.

## AUTHOR CONTRIBUTION

**Zorimar Vilella‐Pacheco:** Conceptualization (lead); Data curation (equal); Formal analysis (equal); Investigation (lead); Methodology (lead); Project administration (equal); Visualization (equal); Writing‐original draft (lead); Writing‐review & editing (equal). **Lisa Mitchem:** Conceptualization (lead); Data curation (supporting); Formal analysis (lead); Investigation (equal); Methodology (equal); Project administration (equal); Visualization (lead); Writing‐original draft (equal); Writing‐review & editing (lead). **Vincent A. Formica:** Funding acquisition (lead); Investigation (supporting); Project administration (supporting); Resources (supporting); Supervision (equal); Validation (equal); Writing‐review & editing (equal). **Edmund D Brodie, III:** Funding acquisition (lead); Resources (lead); Supervision (lead); Validation (lead); Writing‐review & editing (equal).

## Data Availability

All data and relevant R code used are archived in Dryad Repository at https://doi.org/10.5061/dryad.0p2ngf1xt.
